# Variations in Metal Tolerance and Accumulation in Three Hydroponically Cultivated Varieties of *Salix integra* Treated with Lead

**DOI:** 10.1371/journal.pone.0108568

**Published:** 2014-09-30

**Authors:** Shufeng Wang, Xiang Shi, Haijing Sun, Yitai Chen, Hongwei Pan, Xiaoe Yang, Tariq Rafiq

**Affiliations:** 1 MOE Key Lab of Environmental Remediation and Ecosystem Health, College of Environmental and Resource Sciences, Zhejiang University, Zijingang Campus, Hangzhou, P.R. China; 2 Research Institute of Subtropical Forestry, Chinese Academy of Forestry, Fuyang, Hangzhou, P.R. China; University of Vigo, Spain

## Abstract

Willow species have been suggested for use in the remediation of contaminated soils due to their high biomass production, fast growth, and high accumulation of heavy metals. The tolerance and accumulation of metals may vary among willow species and varieties, and the assessment of this variability is vital for selecting willow species/varieties for phytoremediation applications. Here, we examined the variations in lead (Pb) tolerance and accumulation of three cultivated varieties of *Salix integra* (Weishanhu, Yizhibi and Dahongtou), a shrub willow native to northeastern China, using hydroponic culture in a greenhouse. In general, the tolerance and accumulation of Pb varied among the three willow varieties depending on the Pb concentration. All three varieties had a high tolerance index (TI) and EC50 value (the effective concentration of Pb in the nutrient solution that caused a 50% inhibition on biomass production), but a low translocation factor (TF), indicating that Pb sequestration is mainly restricted in the roots of *S. integra*. Among the three varieties, Dahogntou was more sensitive to the increased Pb concentration than the other two varieties, with the lowest EC50 and TI for root and above-ground tissues. In this respect, Weishanhu and Yizhibi were more suitable for phytostabilization of Pb-contaminated soils. However, our findings also indicated the importance of considering the toxicity symptoms when selecting willow varieties for the use of phytoremediation, since we also found that the three varieties revealed various toxicity symptoms of leaf wilting, chlorosis and inhibition of shoot and root growth under the higher Pb concentrations. Such symptoms could be considered as a supplementary index in screening tests.

## Introduction

Heavy metal contamination in water, air, or soil is a major environmental concern worldwide [1]–[3]. Excessive levels of heavy metals can be introduced into the environment by mining, smelting, electroplating, use of pesticides or fertilizers, industrial discharge, etc. [1], [4]–[7]. In China, twenty million hectares of agricultural land have been polluted with heavy metals, and it has been reported that each year over 12 million tons of grain are contaminated by toxic metals [8]. Heavy metals cannot be degraded but can be stabilized or extracted by plants, in a process known as “phytoremediation” [1], [4], [9], [10]. The phytoremediation of heavy metals is affected by several factors, such as the species of plant, solubility in soil solution, and the translocation of the heavy metals from the soil to the harvestable plant parts, etc. [11], [12]. Of these factors, the plant species plays a crucial role, and therefore, selecting appropriate plant species is important for the successful application of plant-based remediation techniques.

Lead (Pb) is one of the most toxic metals, and its concentration in agricultural soil has rapidly increased due to various anthropogenic inputs [13]. Lead is not a bio-essential element, but is easily absorbed by and accumulated in plants [14], [15]. Many studies have shown that the patterns of Pb uptake, transport and accumulation in plants are strongly governed by plant factors, and that Pb accumulation and distribution vary largely among different plant organs [3], [16]. Most studies have shown that, for the majority of plant species, Pb uptake is restricted to roots, with only a small portion being translocated to the shoots [12], [17]–[19]. Whereas hyperaccumulation is an exception, for instance, *Thlaspi rotundifolium*, a cadmium hyperaccumulator, is capable of accumulating up to 8,200 mg kg^−1^ Pb in the shoots [10]. Despite the abilities of these plants to accumulating high amounts of Pb in above-ground tissues, their application in contaminated soil remediation is often limited because of their slow growth rate and low biomass yield [20]. In contrast, other species e.g., *Brassica juncea* (cv.426308) have been shown to accumulate high Pb concentrations in shoots (34,500 mg kg^−1^) and in addition have high biomass which allows high metal removals [12]. However, their growth performance and metal-accumulating abilities can also be limited by low pH and high Pb concentration (>1, 500 mg kg^−1^) in the soil [21].

Plant species are selected for use in the remediation of heavy metal contamination based on factors, such as high biomass production, ability to take up and accumulate metals, deep root systems, high growth rate, and ease of planting and maintenance [1], [22]–[24]. Trees, especially those fast-growing woody species, with their large biomass and deeper, more integrated root systems, have provided a unique means for deep phytoremediation of soil or water during recent decades [11], [25], [26]. In recent years, willow species, which could be grown intensively for use in energy production, have been suggested for use in the remediation of metal contaminated soils. A number of *Salix* species have been studied for their ability to tolerate and accumulate heavy metals [27]–[30]. Ali *et al*. [9] demonstrated that *Salix acmophylla* can accumulate considerable amounts of Cu, Ni and Pb in different plant parts and exhibits high tolerance to these metals. Tlustos *et al*. [30] reported that *S. smithiana* Willd. is able to accumulate 456 mg kg^−1^ and 26.6 mg kg^−1^ Pb in the roots and trunk, respectively, but no more than 10 mg kg^−1^ Pb was observed in the twigs and leaves. Most of the studies confirmed that *Salix* species are able to accumulate a high concentration of Pb in the root systems and have the potential for phytostablization of Pb, and that the uptake patterns and Pb accumulation in *Salix* vary among species and varieties [30], [31]. However, the critical concentration causing Pb-induced phytotoxicity or growth inhibition in different *Salix* species or varieties is still unknown. Hydroponic methods are effective in the rapid screening for heavy metal tolerance and accumulation in plants, and have been widely used in evaluating the phytoremediation potential of willow species in recent years [18], [29].

The aim of our study was to detect the Pb accumulation potential, especially the critical toxicity thresholds based on EC50 estimates in three cultivated varieties of *Salix integra* using hydroponic methods. The results are important for effective selection of willow species for phytoremediation application.

*Salix integra*, a shrub willow native to northeastern China, Japan, Korea, and Primorsky Krai in the far southeast of Russia, has been identified as a Cd-accumulating plant [32], [33]. Cultivation and planting of *S. integra* for shoot and biomass production have a long history in China, and many cultivated varieties are grown in northeastern China. Yizhibi, Dahongtou and Weishanhu are three cultivated varieties of *S. integra* with high biomass production and easy cultivation [34]–[36]. In previous studies, we have confirmed that *S. integra* was able to tolerate and accumulate high concentrations of Cd and Zn in hydroponic culture, and we measured their differences in Cd uptake and accumulation, as well as the tolerance indices, in the *S. integra* varieties [35]–[37].

In the present study, we hypothesized that the three varieties of *S. integra* could take up and accumulate Pb in their roots and above-ground parts, and that Pb accumulation would vary among the varieties according to the Pb contamination level. To test this hypothesis, hydroponic culture experiments were conducted to compare the Pb tolerance and accumulation among the three varieties of *S. integra* and to determine the critical level of Pb for their normal growth. The results are expected to improve our understanding of metal accumulation patterns in *Salix* species and provide guidance for future application of *S*. *integra* in the phytoremediation of Pb contaminated soils and/or water.

## Materials and Methods

### Experimental site and willow preparation

The experiment was conducted in a greenhouse in Fuyang, Hangzhou, Zhejiang Province, P. R. China (30°03′N, 119°57′E) in May, 2011. The greenhouse temperature was maintained between 20–25°C, with a natural photoperiod. The *S. integra* varieties Weishanhu, Yizhibi and Dahongtou were obtained from native habitats in Shandong Province. No specific permits were required to extract samples from this site, which is not privately-owned, and this study did not involve protected species. Cuttings (8–10 cm) from 1-year-old stems in nursery beds at the Institute of Subtropical Forestry were selected for uniformity based on the diameter (Φ 0.4–0.5 cm) and the number of buds (4–6 buds per cutting). Cuttings were rooted in tap water for 4 wk in hydroponic pots (50 cm×35 cm×15 cm, length × width × height) and then transferred to 15 L aerated Knop's solution [38] (6.1 mM Ca (NO_3_)_2_, 2.5 mM KNO_3_, 1.6 mM KCl, 1.8 mM KH_2_PO_4_, 2.1 mM MgSO_4_ and 3.8 µM FeCl_3_) in each pot, maintaining a constant pH of 5.5 using 1 M HCl or 1 M NaOH.

To avoid possible Pb precipitation caused by the presence of phosphorus (P) in the nutrient solution, the P concentration was kept at a maximum of 0.04 µM according to Zhivotovsky *et al*. [18].

### Experimental design

After 2 wk of plant growth in the aerated solution, Pb treatments with the final concentration of 47 µM (T1), 123 µM (T2), 178 µM (T3), and 196 µM Pb (T4) were applied as Pb(NO_3_)_2_ for 14 days, with normal nutrient solution as the control. Each treatment was replicated three times, with each replicate consisting of one pot containing six plants for each variety. These pots were arranged in a completey randomized block design.

The metal and nutrient solutions were replaced every 2 d to ensure consistency. Metal-related phytotoxicity symptoms were recorded throughout the experiment. At the end of the growth and Pb treatment period, the plant tissues were separated into roots, wood (the original cuttings), new shoots, and leaves, washed with tap water and rinsed with deionized water. The root length, surface area, volume, diameter and number of root tips of each plant were determined using root scan apparatus (Epson V700), equipped with WinRHIZO software (Regent Instruments Co.). The root tissues were then washed in 1 M HCl solution and rinsed again with deionized water. Samples were dried at 70°C for 48 h and the dry weights were recorded.

### Estimation of chlorophyll content

As a non-destructive measurement, we used an Opti-Sciences CCM-200 chlorophyll-meter to estimate the chlorophyll content by recording the Chlorophyll Concentration Index (CCI).

### Pb analysis in plant tissues

All plant tissue samples were ashed at 500°C in a muffle furnace, and the ash was dissolved in 10 ml of 1 M HCl solution, and diluted in double deionized water to a 50 ml volume prior to analysis. The total Pb concentration in the liquid samples was determined using inductively coupled plasma optical emission spectrometer (Varian 725-ES, Palo Alto, CA, USA). Certified reference materials (Mixed shoots of shrubs from Pb-Zn mine tailings, GBW 07602, China) were used to ensure the quality of analyses. Good agreement was obtained between our method and certified values. The total metal contents (mg plant^−1^) in the roots and above-ground tissues (wood, new shoots, and leaves) were calculated by multiplying the tissue dry weight by the metal concentration.

### Determination of tolerance index

The tolerance index (TI) was determined to assess the ability of the willow varieties to grow in the presence of a given concentration of Pb according to the following equation [39]:
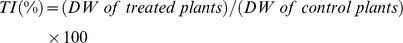
where DW is the dry weight of the roots or above-ground tissues of the willow.

### Determination of translocation factor

The translocation factor (TF) indicates the efficiency of the plant to translocate the accumulated metal from its roots to the aerial parts. It is calculated as follows [40]:
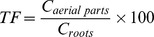
where *C_aerial_* parts is the concentration of metal in the above-ground tissues and *C_roots_* is the concentration of metal in the roots.

### Determination of EC50

The EC50 (effective concentration) is the concentration of the metal that causes a 50% decrease in plant biomass, as compared with the control. Critical toxicity thresholds based on EC50 estimates were determined for the roots and above-ground tissues (combined wood, new shoots, and leaves) for each variety using non-linear regressions to fit curves [41]. A multivariate model using different variances was used to compare the EC50s among willow varieties [42]:
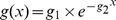
where “*g*” is the measured biomass. “*g_1_*” and “*g_2_*” are the parameters to be estimated and “*x*” is the treatment.

### Statistical methods

Data were analyzed using the statistical package Data Processing System (DPS 13.01) [43], Origin7.5 and Excel 2003 for Windows. All data were tested for homogeneity of variance and normality. Differences among treatments and cultivars were analyzed by one-way or two-way ANOVA (*P*<0.05) according to Fisher's LSD test.

## Results

### Visual symptoms

**Figure 1:**
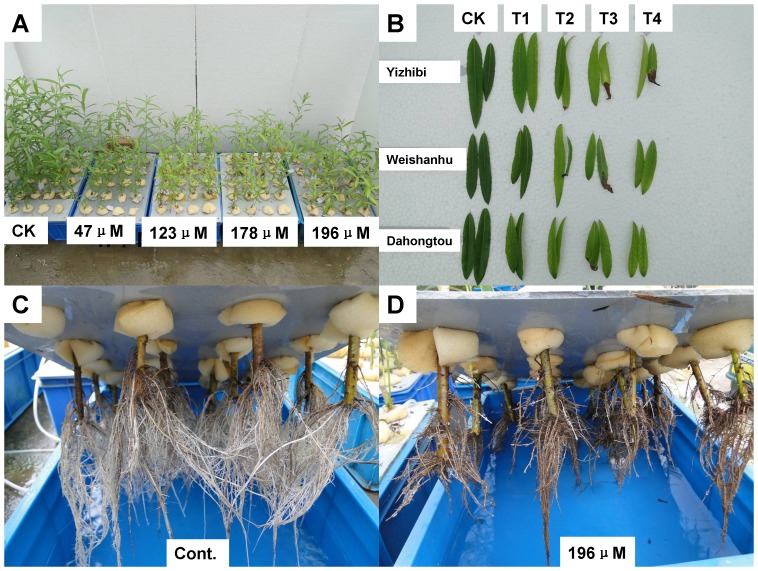
Growth development of *S. integra* exposed to different Pb concentrations for 14 days and metal related symptoms in roots and leaves. (A) Growth of *S. integra* after exposure to 0, 47, 123, 178, and 196 *µ*M of Pb treatments for 14 days. (B) Leaf symptoms of three varieties under different Pb treatments for 14 days. T1, T2, T3 and T4 represent 47, 123, 178, and 196 *µ*M of Pb respectively. (C) Root systems of willows grown in the control. (D) Root systems of willows grown under 196 *µ*M Pb treatment.

After 5 d of Pb exposure, the foliage of willow plants treated with 123,178 and 196 µM began to yellow and wilt from the leaf tip. Weishanhu treated with 123 µM Pb exhibited the earliest leaf wilting. Dahongtou exhibited more pronounced symptoms, including interveinal chlorosis of the basal leaf part, which became more severe on the day of harvest (Fig 1B). The Chlorophyll Concentration Index (CCI) at harvesting was significantly lowered (*P*<0.05) by Pb in all three varieties, and no significant differences were observed among the three varieties (Table 1). In addition, the willow plants had smaller leaves with higher Pb concentrations (178 and 196 µM Pb) (Figure 1B).

**Table 1:** Chlorophyll Concentration Index (CCI) of leaves of three varieties of *S. integra* grown in various Pb concentrations for 14 days.

	Varieties		
Pb (µM)	Yizhibi	Weishanhu	Dahongtou
0	13.05±0.93a	13.77±0.16a	11.79±1.14a
47	8.50±0.53b	8.84±0.53b	8.40±0.08b
123	5.58±0.23c	6.39±1.01c	6.20±1.47c
178	6.13±0.78c	6.18±0.68c	6.13±0.72c
196	4.78±0.13c	5.43±0.16c	5.27±0.13c

Values are mean ± S.D. (n = 6), and data with different letters in the same column indicate a significant difference at *P*<0.05 according to Fisher's LSD test.

After 14 d of Pb treatment, the shoot growth was significantly reduced (Figure 1A) in all varieties for all Pb treatments, compared with the control, but no significant differences were observed among the 47, 123, 178, and 196 *µ*M Pb treatments.

**Figure 2:**
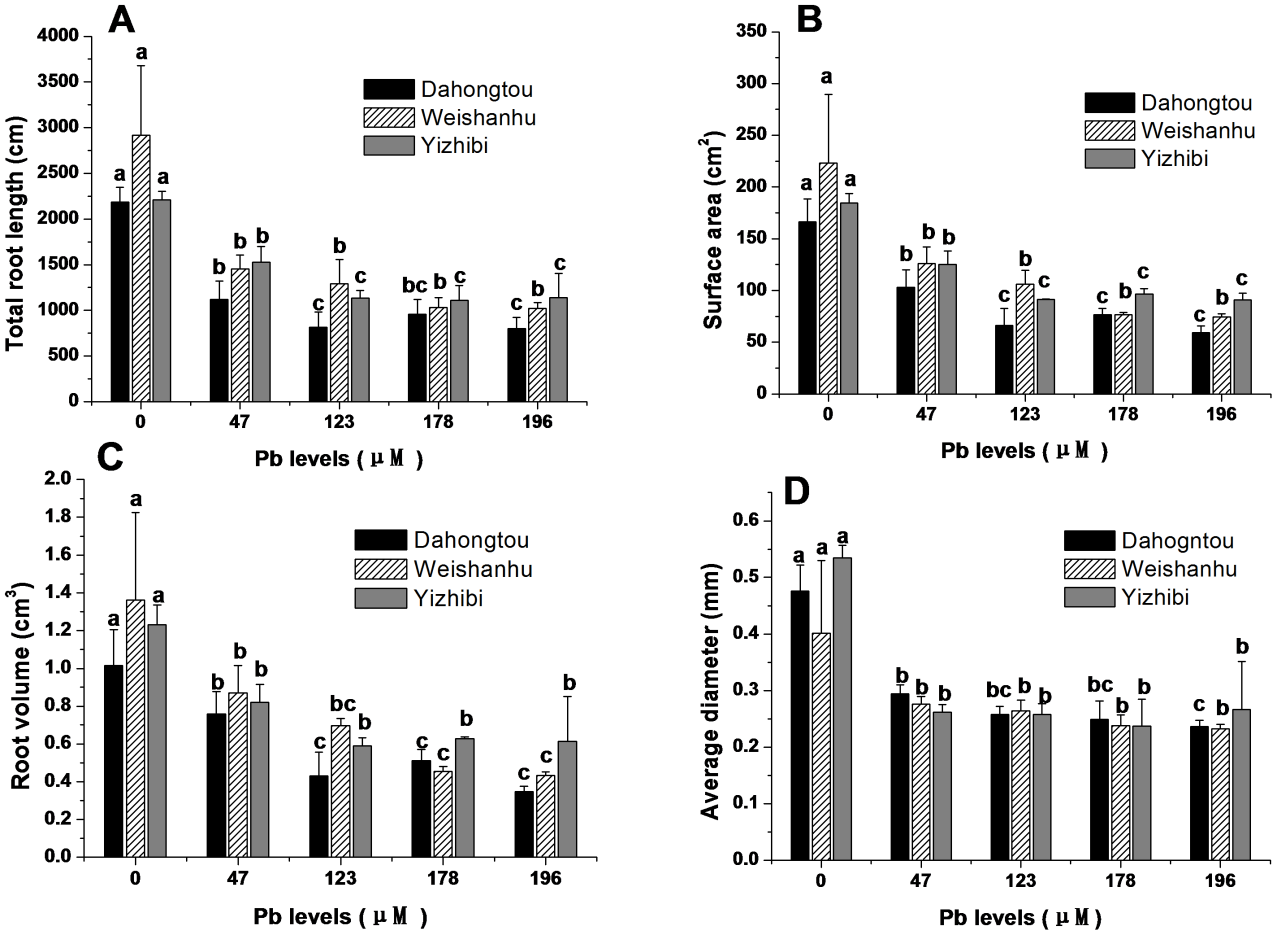
Root characteristics of three *S. integra* varieties exposed to different Pb concentrations for 14 days. (A) Total root length. (B) Surface area. (C) Root volume. (D) Average diameter. Data points and error bars represent means ± S.D. of three replicates (*n* = 3). Different letters indicate significant differences (*P*<0.05) across the treatments according to Fisher's LSD test.

The roots exhibited different levels of blackening and their growth was stunted after exposure to Pb regardless of the Pb concentration, or willow variety (Figure 1C, D). The symptoms became more severe with increasing Pb concentrations. However, no significant differences were observed among the three varieties of *S. integra*. Besides blackening, the total root length and surface area of three varieties were also reduced significantly by the Pb treatments (*P*<0.05), but the reduction was more pronounced in Dahongtou than in Weishanhu or Yizhibi (Figure 2A, B). There was a similar reduction in root volume to that observed in the total root length and surface area in the three varieties (Figure 2C). The root diameters were significantly reduced by the Pb treatments (Figure 2D), but there were no significant differences among varieties or Pb levels.

### Biomass production and tolerance Index

The dry weights of the roots and above-ground tissues (wood, new shoots, and leaves) of *S. integra* varieties are presented in Table 2. The largest root and above-ground tissue biomass for the Pb treatments were recorded for Weishanhu (0.12 and 1.66 g plant^−1^ respectively). Compared with the control, the dry weight of the above-ground tissues of three varieties reduced significantly (P<0.05) at higher Pb treatment (178 and 196 µM). While at lower Pb treatment (47 µM), no significant differences were observed in the dry weight of the above-ground tissues of Weishanhu and Yizhibi, whereas there was a significant (P<0.05) reduction in that of Dahongtou under the same Pb treatments.

**Table 2:** Dry weight (DW) (g) of root and aboveground tissues (combined wood, new shoots and leaves) and shoot length (cm) of three willow varieties grown in various Pb concentrations for 14 days.

	Varieties		
Pb treatment (µM)	Yizhibi	Weishanhu	Dahongtou
DW of Aboveground tissue (g)			
0	1.64±0.10ab	1.88±0.27a	1.90±0.07a
47	1.70±0.19a	1.66±0.13ab	1.50±0.15b
123	1.38±0.12bc	1.62±0.08abc	1.46±0.25bc
178	1.43±0.10cd	1.56±0.09bc	1.35±0.09bc
196	1.15±0.14d	1.36±0.09c	1.20±0.09c
DW of Root (g)			
0	0.093±0.007a	0.12±0.01a	0.10±0.01a
47	0.083±0.013a	0.12±0.03a	0.08±0.00a
123	0.052±0.003b	0.08±0.01b	0.06±0.01b
178	0.057±0.003b	0.07±0.01b	0.06±0.00b
196	0.062±0.007b	0.08±0.02b	0.05±0.01b
Shoot length (cm)			
0	40.03±1.15a	45.34±1.77a	39.94±4.73a
47	31.47±0.79b	32.37±3.22b	28.97±0.87b
123	31.31±3.30b	31.67±1.17b	28.72±1.68b
178	28.39±2.36bc	20.56±9.85bc	27.78±3.50b
196	25.81±3.64c	26.28±2.01c	17.17±3.33c

Values are mean ± S.D. (n = 6), and data with different letters in the same column indicate a significant difference at *P*<0.05 according to Fisher's LSD test.

For all varieties, there were no significant differences in the dry weight of the roots between the control and the 47 µM Pb treatment, whereas a significant decrease (*P*<0.05) in root dry weight was observed in all three varieties exposed to Pb concentrations of 123,178 and 196 µM, compared with the control. However, the root biomass of three varieties did not change significantly across the Pb concentraitons.

Shoot growth was significantly inhibited (*P*<0.05) by Pb addition in all the varieties especially at the highest Pb concentration. The longest shoot length for all the treatments, including the control, was measured in Weishanhu (45.34 cm).

The TIs observed in each variety for the different Pb concentrations are listed in Table 3. For Yizhibi and Weishanhu, the TIs decreased significantly at 196 µM Pb, whereas at 123 and 178 µM Pb, they were only slightly reduced compared to the treatment with 47 µM Pb. For Dahongtou, there were no significant differences in TI among the treatments. The TI varied among the three varieties under the different treatments. Weishanhu had the highest TI at all Pb concentrations, except at 47 µM, when the highest TI was measured for Yizhibi.

**Table 3:** Tolerance index (TI) in three *S. integra* varieties after 14 days of exposure to increasing concentrations of Pb.

	Varieties		
Pb treatment (µM)	Yizhibi	Weishanhu	Dahongtou
47	95.80±4.50a	89.20±5.90a	79.91±10.04a
123	81.37±9.09ab	85.83±9.38ab	76.69±15.92a
178	80.92±1.76ab	82.72±12.89ab	70.43±2.32a
196	66.87±9.72b	73.15±15.83b	62.78±2.54a

Values are mean ± S.D. (n = 6), and data with different letters in the same column indicate a significant difference at *P*<0.05 according to Fisher's LSD test.

### Toxicity thresholds

The Pb toxicity thresholds of the three *Salix* varieties were determined for the roots and above-ground tissues (Table 4). All EC_50_ values were >200 µM with the highest EC_50_ value recorded in the roots (258.5 µM) and above-ground tissues (537.5 µM) of Weishanhu. Dahongtou had the lowest EC_50_ values in these tissues. For all three varieties, the EC_50_ values in the roots were lower than those in the above-ground tissues (*P*<0.05).

**Table 4:** Calculated EC50 toxicity thresholds, models, R and P. for roots and the aboveground tissues (combined wood, new shoots and leaves) of three willow varieties exposed to increasing levels of lead.

*S. integra* varieties	Model	R	P	EC50 (µM)
Roots				
Yizhibi	*y* = 0.091727*e ^−0.002786*x*^	0.8925	0.0416	242.6
Weishanhu	*y* = 0.123656*e ^−0.002779*x*^	0.9566	0.0108	258.5
Dahogntou	*y* = 0.095516*e ^−0.003416*x*^	0.9897	0.0013	204.5
Aboveground tissue				
Yizhibi	*y* = 1.7132*e ^−0.001531*x*^	0.8685	0.0561	481.3
Weishanhu	*y* = 1.8468*e ^−0.001252*x*^	0.9132	0.0303	537.5
Dahogntou	*y* = 1.8086*e ^−0.001921*x*^	0.9298	0.0221	335.9

### Metal concentration in plant tissue and translocation factor

The Pb concentrations in the roots, wood, new shoots and leaves varied significantly (*P*<0.05) across treatments, but no significant differences were observed among the varieties, with the exception of wood. For the roots and leaves, there were significant differences (*P*<0.05) in Pb concentration across the varieties × treatment interactions (Table 5).

**Table 5:** Two-way ANOVA analysis for Pb concentration in root, new shoots, wood, and leaves.

Variable	root		New shoots		wood		leaves	
	F	P	F	P	F	P	F	P
Variety	1.04	0.3960	3.22	0.0942	5.81	0.0277	0.60	0.5697
Treatment	19.64	0.0003	14.52	0.0010	82.81	0.0000	7.87	0.0071
Variety × treatment	5.67	0.0003	1.65	0.1561	1.14	0.3664	9.65	0.0000

Lead concentrations in the control were generally below the detection limit. The highest Pb concentrations were found in the roots for all varieties and ranged from 6,799–24,597 mg kg^−1^ (Table 6). All varieties had the lowest Pb concentration in their roots with the 47 µM Pb treatment, ranging from 6,799–9,632 mg kg^−1^. There was a steady increase in the root Pb concentration in Yizhibi and Dahongtou with increasing Pb treatment concentration, and a significant increase (*P*<0.05) was observed in all varieties at 47 µM Pb treatment. The Pb root concentration in Weishanhu increased significantly with 123 µM Pb, whereas decreased when the Pb treatments ≥123 µM. No significant differences in the root Pb concentration of the three varieties were observed between the 178 µM and 196 µM Pb treatments.

**Table 6:** Average Pb concentrations (mg kg^−1^) in dry plant tissues of *S. integra* exposed to various Pb treatments for 14 days.

		Varieties		
Plant tissue	Pb µM	Yizhibi	Weishanhu	Dahongtou
Leaves	0	ND	ND	ND
	47	29.3±11.6b	27.7±5.4a	20.0±8.4b
	123	44.9±7.5a	36.9±9.4a	89.3±19.6a
	178	15.4±4.7c	14.3±3.6b	16.7±5.8bc
	196	13.7±2.7c	9.9±3.8b	8.6±3.8bc
New shoots	0	ND	ND	ND
	47	29.8±9.3a	33.4±4.7a	36.1±10.4a
	123	17.2±1.7a	14.6±2.9b	27.4±10.5a
	178	19.7±9.8a	43.0±7.2a	34.1±1.1a
	196	25.9±8.5a	34.1±13.1a	49.4±23.5a
Wood	0	ND	ND	ND
	47	244.±82.4c	216.0±37.5c	328.0±118.3c
	123	548.3±147.8b	470.3±49.7b	574.0±114.2b
	178	765.0±147.5a	655.0±117.9a	788.7±135.1ab
	196	865.0±69.1a	611.0±64.6a	930.3±199.5a
Root	0	ND	ND	ND
	47	9,632.3±2,268.6b	6,799.7±1,918.1c	8,318.7±1,827.6c
	123	18,271.0±3,334.5a	19,629.0±1,799.5a	13,999.0±9,64.2b
	178	18,074.7±4,816.4a	15,340.7±3,954.3ab	23,998.0±2,760.0a
	196	21,471.0±1,274.9a	14,391.7±3,738.0b	24,597.0±1,855.1a

Values are mean ± S.D. (n = 6), and data with different letters in the same column indicate a significant difference at *P*<0.05 according to Fisher's LSD test.

The Pb concentration in the wood tissue for all the varieties and treatments ranged from 216–930 mg kg^−1^, with the highest Pb concentration displayed by Dahongtou with the 196 µM Pb treatment. With increasing Pb concentration, the wood tissue Pb concentration significantly increased (*P*<0.05) in all three varieties.

The shoot Pb concentration in all three varieties increased significantly (*P*<0.05) under all Pb treatments compared with the control, however, no significant differences were observed among treatments except in Weishanhu, whose shoot Pb concentration decreased significantly with the 123 µM Pb treatment. The highest Pb concentration in new shoots was observed in Dahogntou (49.4 mg kg^−1^) under 196 µM Pb, while the lowest was observed in Weishanhu (14.6 mg kg^−1^) under 123 µM Pb.

The highest Pb concentrations in the leaves for all varieties were observed with treatment of 123 µM Pb. The leaf Pb concentrations declined significantly (*P*<0.05) with increasing Pb treatment concentration.

**Figure 3:**
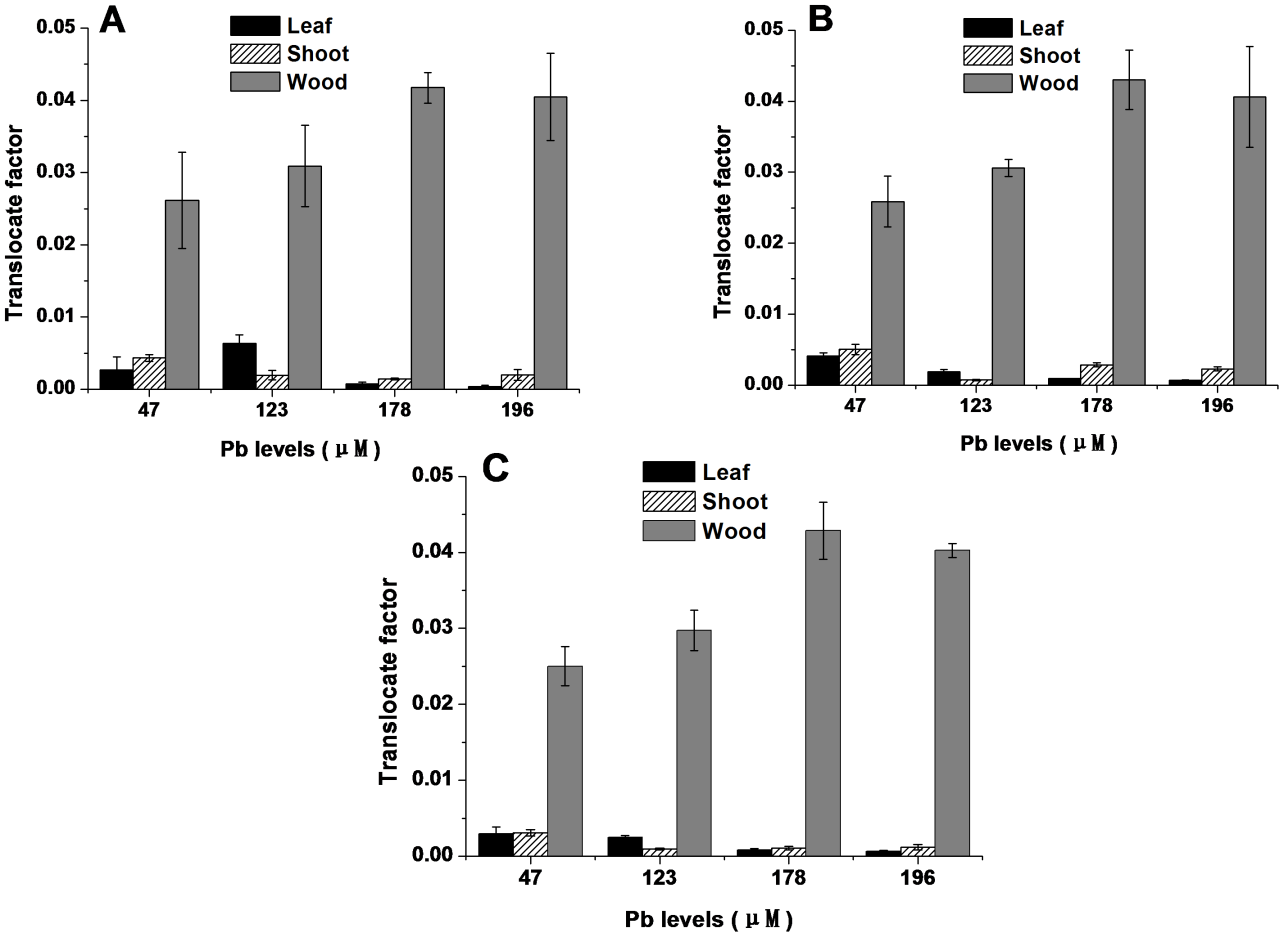
Translocation factor (TF) of leaf, new shoots and wood in three varieties of *S. integra* for the different Pb treatment. (A) Dahongtou. (B) Weishanhu. (C) Yizhibi.

The TF can be used to evaluate the capacity of a plant to translocate heavy metals from the roots to the harvested parts. There were significant (*P*<0.05) differences in the TFs of three varieties among the different tissue types and among different Pb treatments. For all the varieties, the TFs of wood were much higher than those of leaf or new shoots, and increased gradually with increasing Pb treatment concentration, indicating more effective translocation in *S. integra* from the roots to the wood than to the leaves or new shoots (Fig 3). The TFs of leaves and new shoots were extremely low (<0.05) and decreased with increasing Pb concentration in solutions, with the exception of Dahogntou, which displayed a distinctive increase under 123 µM Pb treatment (Fig 3A).

### Metal contents in above-ground tissues and roots

The Pb concent of the above-ground tissues (the combined wood, new shoots and leaves) varied significantly across the treatments and varieties × treatment interactions (*P*<0.05). The variety × treatment interaction effects were also significant (*P*<0.05) in the roots (Table 7), whereas no significant differences in the Pb concent were observed in the roots or above-ground tissues among the varieties.

**Table 7:** Two-way ANOVA analysis for Pb concent in root and the aboveground tissues.

Variable	root		aboveground	
	F	P	F	P
Variety	0.33	0.7290	5.00	0.0527
Treatment	1.89	0.2327	28.47	0.0006
Variety × treatment	4.44	0.0044	2.92	0.0298

The highest root Pb content in Weishanhu was found in the treatment of 123 µM Pb concentration (Table 8), whereas Yizhibi and Dahongtou had the highest Pb content in their roots with the 178 or 196 µM Pb treatments.

**Table 8:** Lead content (mg plant^−1^) in root and aboveground tissue (wood, new shoots, and leaves) of three *S. integra* varieties exposed to different Pb concentrations for 14 days.

		Varieties		
plant tissue	Pb µM	Yizhibi	Weishanhu	Dahongtou
Root	47	0.85±0.26c	0.77±0.16b	0.69±0.15c
	123	0.95±0.21bc	1.56±0.29a	0.83±0.20bc
	178	1.01±0.21b	1.11±0.22b	1.33±0.13a
	196	1.32±0.17a	1.11±0.38b	1.17±0.17ab
Aboveground tissue	47	0.22±0c	0.23±0.01c	0.33±0.06c
	123	0.50±0.09b	0.52±0.03b	0.63±0.06b
	178	0.70±0.12a	0.68±0.07a	0.74±0.07ab
	196	0.65±0.05ab	0.55±0.04b	0.88±0.13a

Values are mean ± S.D. (n = 6), and data with different letters within the same column indicate a significant difference at *P*<0.05 according to Fisher's LSD test.

The highest Pb contents in the above-ground tissues for both Yizhibi and Weishanhu were displayed under 178 µM Pb treatment, whereas the highest Pb content for Dahongtou was exhibited under 196 µM Pb treatment. All varieties had the lowest Pb content in their roots and aboveground tissues with the 47 µM Pb treatment.

## Discussion

Excess Pb causes a number of toxicity symptoms in plants, and the non-specific symptoms include stunted growth, chlorosis and inhibition of root growth [44]. In our study, at Pb concentrations ≥123 µM, willows exhibited stunted growth, leaf dehydration and chlorosis, and severely reduced root biomass. Similar Pb toxicity symptoms were observed in previous studies [45]–[48]. According to Sharma and Dubey [44], these toxicity symptoms are some of the physiological responses to metal treatments exhibited by plants, because the presence of Pb in the cell, even in small amounts, can potentially cause a wide range of adverse effects on physiological processes, such as inhibition of enzymatic activities, disturbed mineral nutrition, water imbalance, altered hormonal status, and altered membrane permeability.

After exposure to different Pb concentrations, there were significant variations in the dry weights of the three varieties of *S. integra*. In general, the root biomass was significantly lower than the above-ground biomass among the varieties and treatments. The root dry weights of the three varieties did not change significantly at the lowest Pb concentration of 47 µM, while the above-ground biomass decreased significantly at the same concentration. This suggests that the shoot growth of *S. integra* was more inhibited than the root's at lower Pb concentrations. In contrast, in a previous study [18], the roots of *S. lucida, S. serissima, S. sachalinensis L., S. miyabeana L.*, and *S. nigra* clones showed a significant reduction in biomass at a similar concentration of Pb (48 µM). This indicates that there is inter-specific variability in sensitivity to environmental stress such as Pb increasing concentrations in soil solution. The roots are in direct contact with Pb, and provide the primary route for metal ion penetration [49]. Plant roots rapidly respond to absorbed Pb, through a reduction in root growth and changes in branching pattern [44]. In our study, the development of root morphology of the three varieties were significantly inhibited by all Pb treatments, which is consistent with the behavior of other species, e.g., *Picea abies* [50] and *Zea mays* [51]. Studies have shown that the inhibition of root growth under Pb stress is a result of Pb-induced inhibition of cell division in root tips [52]. Weirzbicka [53] reported that the roots of onion (*Allium cepa*) exhibited a reduction in root growth, mitotic irregularities and chromosome stickiness when exposed to different concentrations of Pb nitrate. Yang *et al*. [54] also observed a disturbance in alignment of microtubules in *Oryza sativa* in the presence of Pb, and damage to microtubules is now considered one of the key components of Pb-induced damage in plants [44], [52].

Lead has also been reported to decrease the chlorophyll content by imparing the uptake of essential elements such as Mg and Fe by plants [55], or by increasing chlorophyllase activity in Pb-treated plants [56]. In our study, we also observed severe chlorosis in the leaves of the three varieties of *S. integra* and a significant decrease in the chlorophyll concentration index, however, further study is required to investigate the reasons for these damages in leaves with Pb treatment.

Assessing metal tolerance is of paramount importance when selecting plants for utilization in phytoremediation [40]. To characterize metal tolerance in plants, one of the most common parameters used is the tolerance index (TI) [57]. Willows have shown significant variations in tolerance across species and clones. In previous studies, significant variations in metal tolerance were found among willow species and clones exposed to cadmium, copper, or arsenic [29], [38], [58], [59]. *S. integra* was also confirmed to have a high capacity for cadmium and zinc uptake [32], [33], [35], but no information about the Pb tolerance and accumulation was reported. There are many cultivated varieties of *S. integra* in China, and the three varieties evaluated in this study were chosen because of their different tolerances to Cd treatment [35]–[37]. Although TI varies with increasing Pb concentration, all three varieties of *S. integra* tested can be defined as highly tolerant (TI >60) to Pb according to the scheme proposed by Lux *et al*. [60], with Weishanhu being the most tolerant variety in our study.

The EC50 value is another important parameter that describes the tolerance of plant species in multiple concentration tests [57]. In our study, we observed that the EC50 values of the roots were lower than those of the above-ground parts of the plants, suggesting that the roots of *S. integra* were more sensitive to Pb treatment. A similar behavior was observed in *S. lucida, S. serissima, S. sachalinensis L., S. miyabeana L.*, and *S. nigra clones* [18]. However, more interesting are the extremely high EC50 values for the roots and aboveground tissues of the three varieties which we observed in *S. integra*. Previously, EC50 values for willows in the presence of Pb in hydroponic culture were recorded as 6–32 µM [18], while we measured values >200 µM. High tolerance to metals is a key factor for a plant to successfully colonize metallic soil. Based on the observed EC50 values, Weishanhu was found to be the most tolerant of the three varieties, and could be a suitable candidate for Pb phytostablization in contaminated areas.

A number of studies have shown that Pb accumulates preferentially in roots [19], [61], and our results are consistent with this observation. Sharma and Dubey [44] reported that Pb is confined in roots probably because it binds to ion exchangeable sites on the cell wall, with further extracellular precipitation as Pb carbonates. It has also been reported that Pb has a strong ability to bind to the carboxyl groups of galacturonic and glucuronic acids in the cell wall, which limits the apoplastic transport of this metal [62]. In addition, the Casparian strip of the endodermis plays an important role in restricting Pb transport across the endodermis into other tissues [63]. In addition to the physical barrier that causes poor translocation of Pb from the roots to new shoots, some researchers reported that a short-term exposure to a heavy metal could also result in poor translocation of Pb [64]. Zhivotovsky [18] conducted a short-term hydroponical test to show that *Salix* may need more time to adapt to a specific environment to improve the transport of Pb to above-ground plant tissues. In our study, we also used a short-term screening test, and found Pb accumulation patterns were similar to those of previous studies. However, it is interesting that we observed higher concentrations of Pb in the wood cuttings than in the new shoots and leaves, which showed that Pb absorbed by the roots was transported to the woody parts more readily than to the new shoots and leaves. We also observed that Dahogntou exposed to 123 µM Pb was most efficient in translocating Pb into the leaves. In addition, the highest Pb root concentration we recorded (24,597 mg kg^−1^) was higher than those that have been reported in the literature for *Salix* (4,164–23,023 mg kg^−1^) grown in either soil or solution [18], [65]. Our results indicate that the roots of *S. integra* can both tolerate and accumulate high Pb concentrations; however, the tolerance mechanism in the root system is still unknown. Further research is required to evaluate whether this tolerance to Pb in roots is acquired by blocking the entrance of Pb into the root cells, or by some metabolic mechanisms that detoxify Pb toxicity.

## Conclusions

Willows have potential for phytoremediation due to their high biomass productivity, high transpiration rate, and species-specific heavy metal uptake [28], [66], [67]. *S. integra* is a fast-growing shrub willow, and in China, it is generally cultivated by short-rotation plantation, with the new shoots harvested for use in weaving. Yizhibi, Dahogntou and Weishanhu are three cultivated varieties of *S. integra*, that display excellent in biomass production and environmental adaptation [34], [35]. We demonstrated that *S. integra* can tolerate, transport and accumulate various levels of Pb when exposed to different Pb concentrations, and there is no significant difference in the tolerance to Pb among the varieties. For effective phytoextration, we are interested in plants that exhibit not only high tolerance to heavy metals, but also high accumulation of metals in tissues with high biomass production. We observed that Dahongtou had higher Pb contents in the above-ground tissues and the highest TF in leaves under 123 µM Pb. These results bring new insight into the selection of candidates for Pb phytoextraction. However, the results are based on a short-term study, the potential effectiveness of *S. integra* Dahongtou in phytoextraction should be tested in the long-term and open-field studies. In summary, all the varieties, especially Weishanhu and Yizhibi, have high TI and EC50, demonstrating their high tolerance and lower sensitivity to Pb and, thus, their suitability for phytostabilization of Pb.
